# Supplementary material for: Multi-period uncertain portfolio selection model with prospect utility function
Source: PLoS One. 2022 Sep 14;17(9):e0274625. doi: 10.1371/journal.pone.0274625 (PMC9473444; doi:10.1371/journal.pone.0274625)
Supplement: Attachment — Submitted filename: Response to Reviewers.docx [file pone.0274625.s003.docx]

Dear Editor,

We greatly appreciate the editor’s and reviewers’ insightful review of our manuscript entitled “Multi-period uncertain portfolio selection model with prospect utility function”. The comments have helped us make substantial improvements to this manuscript. Following the Editor’s recommendations along with Referees comments, we have revised the manuscript carefully. The main revisions have been marked in red in the revised manuscript. In addition, we did our best to revise the manuscript and rephrased the duplicate text.

We sincerely hope that contents of this paper in present form would be useful for readers of the PLOS ONE, and revised manuscript will be acceptable to you, Editor and the esteemed referees. This manuscript represents the authors’ original work and has not been published nor has it been submitted simultaneously elsewhere. All authors have checked the manuscript and have agreed to the submission.

Waiting for your early and positive response.

Thanking you

Dr. Gaohuizi Guo

Corresponding Author

**Respond to the Reviewer 1 Comments**

**Comment 1:** This is a detailed paper that proposes a new improved artificial bee colony (ABC) method for multi-period uncertain portfolio selection. The explanation on how to improve it are well presented. However, when compared to other techniques, the choice of ABC is not insufficiently discussed. By introducing CPT for the first time, the abbreviations should be used correctly to improve the article.

**Reply:** Thanks for your suggestions. According to your valuable comments, we have

revised the manuscript carefully. Firstly, we have corrected the abbreviation CPT, which was first introduced on page 8. Second, we compare the ABC algorithm with other techniques on page 9 and explain why we chose the ABC algorithm.

**Respond to the Reviewer 2 Comments**

**Comment 1:** This paper is advised to revise the literature review section, including these related references. The authors can consider including the following research works to enrich the literature study.

(i)[https://www.sciencedirect.com/science/article/abs/pii/S1568494615005955/](https://www.sciencedirect.com/science/article/abs/pii/S1568494615005955/)

(ii) [https://link.springer.com/article/10.1007/s12652-017-0478-4](https://link.springer.com/article/10.1007/s12652-017-0478-4)

**Reply:** Thanks for your suggestions. We have revised the literature review section, added newer references, and enriched the study of the literature. These include the study of solving multi-period portfolio problems and the study of multi-period portfolio optimization problems in uncertain environments. They are marked in red in the text.

**Comment 2:** This paper introduces the ABC algorithm and proposes a novel improved method. However, different algorithms have different applications. The author should introduce the application of the ABC algorithm in the field of the portfolio.

**Reply:** Thanks for your suggestions. We introduce the application of the ABC algorithm in the field of the portfolio on page 10 and introduce the relevant references.

**Respond to the Reviewer 3 Comments**

**Comment 1:** This manuscript describes an improved artifificial bee colony algorithms for a multi-period uncertain portfolio selection problem. Numerical results prove the effectiveness of the algorithm under reasonable assumptions, and data analysis is performed appropriately. It is interesting and organized well. The manuscript can be accepted after correcting grammartical、spelling errors.

**Reply:** Thanks for your suggestions. According to your valuable comments, we carefully checked the entire article for grammatical problems and corrected them one by one, and marked them all in red in the text.
